# Comparison of MMF with prednisone in terms of rejection and duration of activity of transplant in rabbits that underwent retroperitoneal heterotopic heart transplantation

**DOI:** 10.5830/CVJA-2014-032

**Published:** 2015

**Authors:** Fatih Aygün, Duran Efe, Kadir Durgut

**Affiliations:** Department of Cardiovascular Surgery, Faculty of Medicine, Mevlana University, Konya, Turkey; Department of Radiology, Faculty of Medicine, Mevlana University, Konya, Turkey; Department of Cardiovascular Surgery, Faculty of Medicine, Erbakan University, Konya, Turkey

**Keywords:** heart transplantation, mycophenolate mofetil, methylprednisolone

## Abstract

**Aim:**

In this study, mycophenolate mofetil (MMF) and methylprednisolone (MP) were compared in terms of rejection and duration of activity of the transplant in New Zealand rabbits that underwent retroperitoneal heart transplantation.

**Methods:**

Retroperitoneal heart transplantation was performed in New Zealand white rabbits. The animals were divided into two groups. MMF group (group 1) (10 donors, 10 recipients): 12.5 mg/kg MMF was administered orally for two days prior to the surgery; MP group (group 2) (nine donors, nine recipients): 2 mg/kg MP was administered intramuscularly for two days prior to the surgery. After the operation, we waited until all motor activity in the transplanted heart had stopped. The transplant was then removed and the recipient was sacrificed. A donor in the MP group was excluded since it died before the motor activity had stopped.

**Results:**

No statistically significant difference was found between the groups in terms of rejection score (*p* = 0.865). However, duration of motor activity was found to be statistically significantly longer in the MMF group, compared to the MP group (*p* = 0.013).

**Conclusion:**

In this experimental study, MMF was similar to MP in terms of rejection but had better efficacy in terms of duration of motor activity of the transplant.

## Abstract

Besides the bicaval anastomosis technique developed in recent years, orthotopic heart transplantation has been successfully performed in the treatment of thousands of patients with heart failure using the surgical technique defined in 1960 by Lower and Shumway.^1^ Heart transplantation has become more common along with advances in preserving organs, illumination of the immunological basis of transplantation, and the constitution of organ transplantation centres to obtain and share organs. The results of experienced centres are similar because heart transplantation has not developed substantially since the 1990s.^2^

Basic problems in the last decade include long waiting lists and extended waiting periods, increased numbers of emergent and pre-emptor patients, and prolonged duration of donor ischaemia. Today, although one-year life expectancy has been reported to be higher than 85%, and 10-year life expectancy 50–60% in the majority of heart surgery centres, it is estimated that the parametric curve will rise to 75% in the next decade along with advances made in recent years.^3^-^5^

Survival after heart transplantation has been extended and substantial progress has been made in heart transplantation practices along with the discovery of immunosuppressive agents. However, the side effects of these immunosuppressive agents and the presence of coronary lesions in the transplanted graft due to extended survival times have become a problem. Reduction in the number of side effects and prevention of the development of vascular lesions in transplanted hearts have been the target of new-generation immunosuppressive agents.

Mycophenolate mofetil (MMF) is under investigation in terms of its effect on vascular lesions and survival in transplanted hearts, as well as non-cardiac transplantation and paediatric cardiac surgery.^1^,^6^ In the present study, the effect of MMF versus methylprednisolone (MP) on acute rejection and duration of motor activity in the transplant was investigated in a rabbit model of retroperitoneal heterotopic heart transplantation.

## Methods

In this study, 38 New Zealand rabbits weighing between 2 550 and 3 200 g were used. The study was conducted in accordance with the ethical committee directive for experimental animals of the Faculty of Medicine, Selçuk University, Meram and the Experimental Medicine Research and Practice Centre, which was prepared based on the Universal Declaration on Animal Welfare, European Convention for the Protection of Vertebrate Animals Used for Experimental or Other Scientific Purposes, and The Guide for the Care and Use of Laboratory Animals. In addition, the study was conducted with the approval of the ethics committee.

Two groups were created; the MMF group: group 1 (donors = 10, recipients = 10) and the MP group: group 2 (donors = nine, recipients = nine). Weights of the rabbits in the MMF group varied between 2 550 and 3 200 g, whereas the weights in the MP group varied between 2 560 and 3 150 g. The two groups were divided into two subgroups, donor and recipient, for retroperitoneal heterotopic heart transplantation.

The subjects of the MP recipient group received 10 mg/kg/day methylprednisolone intramuscularly for two days prior to the surgery (except for the day of surgery). Subjects of the MMF recipient group received 12.5 mg/kg/day orally via the gavage method for two days prior to the surgery (except for the day of surgery).

Intramuscular ketamine hydrochloride (50 mg/kg) (Ketalar®, Phizer) and xylazine (10 mg/kg) (Xylazinbio® 2%, Bioveta) were administered to the animals. The dose was repeated as a cocktail containing ketamine (25 mg/kg) and xylazine (5 mg/kg) when necessary. After anaesthesia, the animals were left to breathe spontaneously and were provided with nasal oxygen (O_2_) support at a dose of 2 l/min.

An intravenous catheter (24-gauge) was placed in each recipient through the marginal ear vein. Over the course of the procedure, 0.9% sodium chloride (NaCl) solution was infused at a speed of 4 ml/kg/hour. A catheter (22-gauge) was placed into the ear artery to monitor blood pressure. The anterior thoracic area and anterior abdominal wall of the recipient was shaved, electrocardiography was performed with electrodes placed on the anterior thoracic wall, and blood pressure was monitored by connecting the catheter placed into the ear artery to the pressure transducer (Mennen Medical Inc, Mercury, Revohot, Israel).

The recipient was continuously monitored during abdominal exploration before the retroperitoneal heterotopic heart transplantation, during transplantation, and after transplantation. Systolic and diastolic blood pressures of the recipients in both groups were kept at the same level as pre-operative measurements. Positive inotropic support was provided as required.

The recipient was placed on the operation table in a supine position. We planned to monitor the recipients for a maximum of four hours and then sacrifice. The abdomen was accessed through a median abdominal incision after monitoring and stabilising the recipients. The retroperitoneum was opened and the inferior vena cava and abdominal aorta were exposed. These two vascular configurations were explored and reversed with the use of tapes. Anticoagulation was provided with 100 U/kg of standard heparin (Nevparin®, Mustafa, Nevzat).

Meanwhile, the donor subject was stabilised in a supine position and anticoagulation was provided with 100 U/kg of standard heparin. After sternotomy the donor heart was excised and crystalloid cardioplegia was administered through the aortic root. Four mini vascular clamps were placed in the recipient’s abdominal aorta and inferior vena cava to prevent blood flow to the anastomoses.

Cold Hospira’s cardioplegia solution (Plegysol®, Meditera) was given through the ascending aorta of the donor’s heart according to the weight of the donor and at the appropriate pressure as soon as the vascular configurations were cut. The time between cutting the ascending aorta of the donor heart and administration of cardioplegia did not exceed 30 seconds in any of the groups. Cardioplegia pressure was kept at 15 mmHg.

After the heart became plegic, the superior vena cava (SVC), inferior vena cava (IVC), and the left atrium were ligatured. The total duration of ischaemia was between 30 and 35 minutes in all subjects, and the second cold crystalloid cardioplegia was administered at the 20th minute. The target was perfusion of the whole heart and passive working of the left heart, whereas the working right heart was filled with blood.

**Figure 1.**
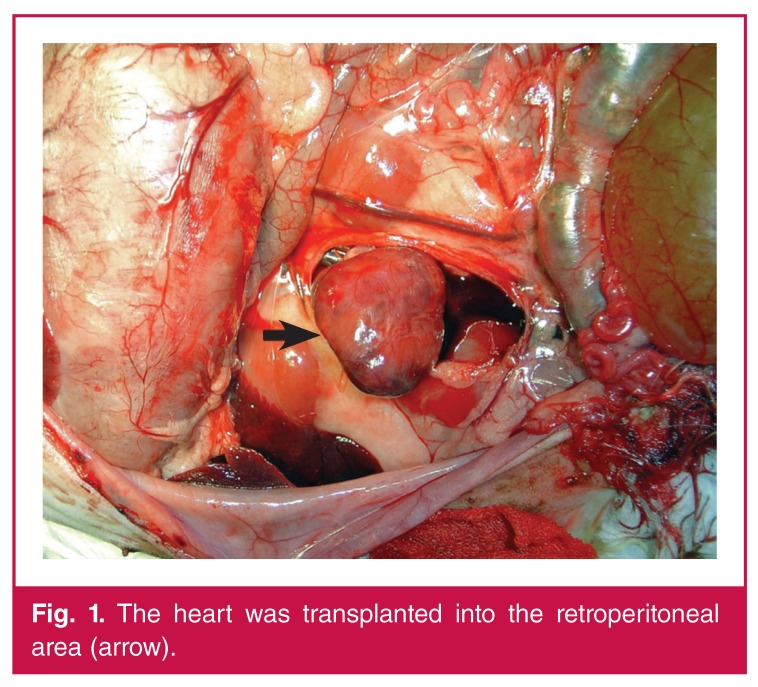
The heart was transplanted into the retroperitoneal area (arrow).

Anastomosis was performed between the ascending aorta of the transplant and the abdominal aorta of the recipient, and between the pulmonary artery of the transplant and the IVC of the recipient. Anastomosis was performed using 7/0 polypropylene suturing material. After transplantation, the vascular clamps in the abdominal aorta and the IVC of the recipient were removed (Fig. 1). The transplant worked spontaneously in sinus rhythm in all experimental groups.

The heart, which was taken from the donor and retroperitoneally implanted in the recipient, functioned for between 2.5 and four hours in all subjects. Systolic and diastolic blood pressures of the recipients were kept the same as pre-operative values as far as possible. Dopamine hydrochloride (Dopamine®, Fresenius) and dobutamine (Dobutabag®, Baxter) were used as positive inotropic support and isotonic 0.9% NaCl solution was used for fluid replacement. After the abdominal aorta and IVC of the recipient were clamped, the heart implanted in the recipient was excised from the anastomosis lines when it stopped functioning.

All recipient subjects were sacrificed at the end of a minimum of 2.5 hours and a maximum of four hours after the activity of the transplant had stopped, and the transplant was removed. Sacrificing was performed using 10% intracardiac formaldehyde after ketamine (50 mg/kg) and xylazine (10 mg/kg) administration via the intramuscular route.

## Histopathological evaluation

The excised transplant was put into 10% neutral formaldehyde solution and stored until examination. Sections were made of the endocardium and myocardium of the right ventricle. After staining with haematoxylin and eosin, the pathologist from the Department of Pathology, SUM Faculty of Medicine, who was blinded to the groups, examined four different areas under a light microscope. Sections were also taken from the rejected transplants of the recipients, stained with haematoxylin and eosin dye and examined under the light microscope.

**Figure 2.**
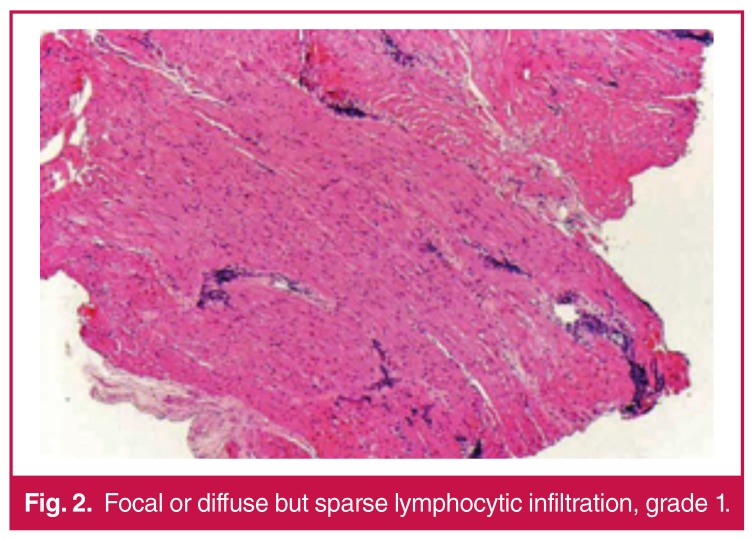
Focal or diffuse but sparse lymphocytic infiltration, grade 1.

**Figure 3.**
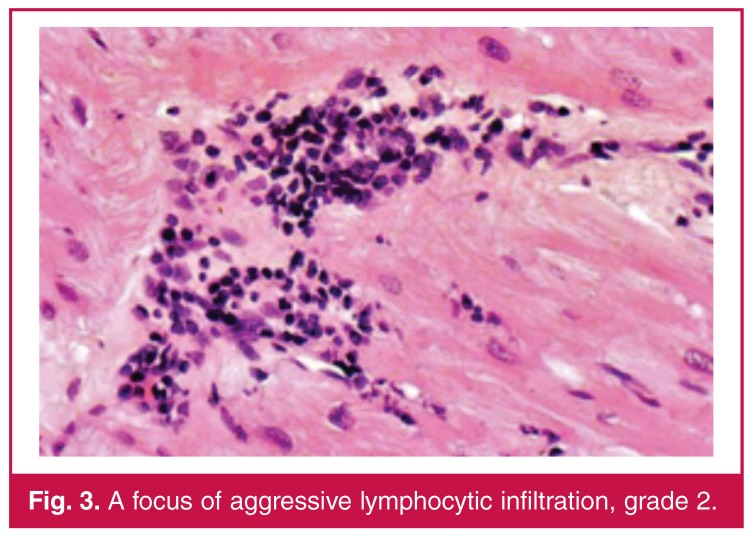
A focus of aggressive lymphocytic infiltration, grade 2.

**Figure 4.**
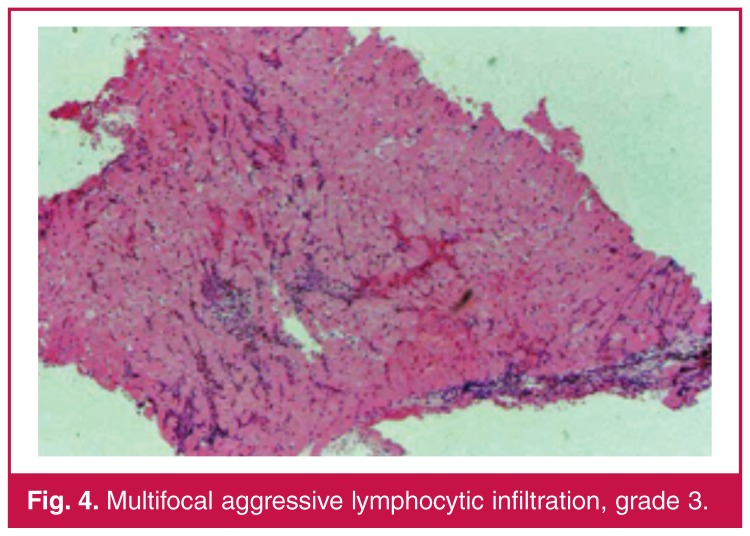
Multifocal aggressive lymphocytic infiltration, grade 3.

**Figure 5.**
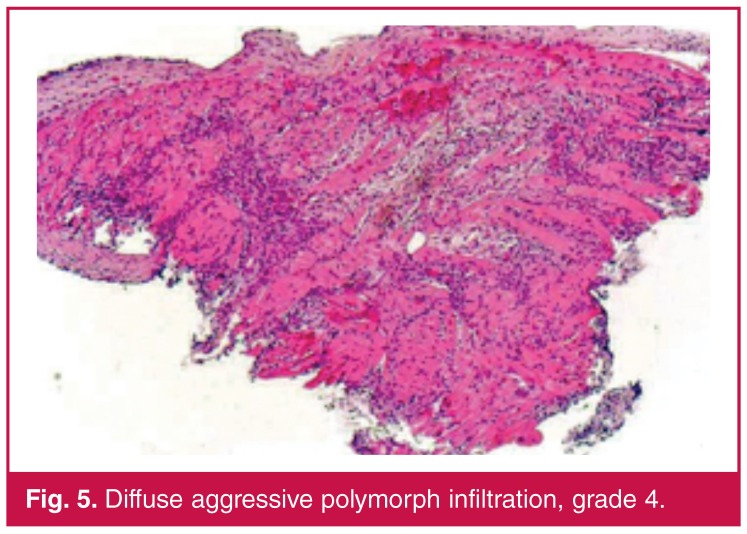
Diffuse aggressive polymorph infiltration, grade 4.

Histological findings on the endomyocardial sections were graded on the basis of the endomyocardial biopsy grading scheme, which is standardised by the International Society for Heart and Lung Transplantation (ISHLT). Grading was defined as follows: no lymphocytic infiltration: grade 0; focal or diffuse but rare lymphocytic infiltration: grade 1 (Fig. 2); unifocal aggressive lymphocyte infiltration: grade 2 (Fig. 3); multifocal aggressive lymphocyte infiltration: grade 3 (Fig. 4); diffuse aggressive polymorph infiltration: grade 4 (Fig. 5).

## Statistical analysis

Data were transferred into the computer. Statistical analyses were done using the SPSS program (SPSS Inc, Chicago, IL, USA). Since the number of subjects in the groups was not equal, they were compared by the Mann–Whitney *U*-test. Level of statistical significance was considered to be *p* < 0.05.

## Results

A total of 19 pairs of New Zealand rabbits were used in this study. The weight of the study subjects was a minimum of 2 550 g and a maximum of 3 200 g. Duration of motor activity after transplant was determined to be a minimum of 2.5 hours and a maximum of four hours in all subjects. Biopsy scoring was found to be a minimum grade 1 and maximum grade 4. Duration of motor activity of the transplants and biopsy scoring of the groups are shown in Table 1.

**Table 1:** Duration of motor activity of the transplants, and biopsy scoring of the groups

	*Group 1 (MMF) (recipient) (n = 10)*	*Group 2 (MP) (recipient) (n = 9)*	p*-value*
Motor activating time (h)	3.20 ± 0.42	2.77 ± 0.26	0.013*
Biopsy scoring	2.80 ± 1.23	2.78 ± 0.83	0.865*
Biopsy scoring (grade)	2761 ± 196.1	2868.3 ± 202.2	

*p-value was presented as a result of Mann–Whitney *U*-test.

## Discussion

In this study, a total of 19 transplantations were performed, of which 10 were in the MMF and nine in the MP group. We aimed to compare MMF, an immunosuppressive agent, with MP, a steroid. Either MMF or MP was administered to the recipients for two days prior to the surgery. The immunosuppressive agent was not given to the subjects on the day of surgery.

The transplant, which was placed retroperitoneally, was excised after its motor activity had completely stopped. It was observed that duration of motor activity of the transplant was statistically significantly longer in the MMF group. No statistically significant difference was observed between the MMF and MP groups in terms of transplant rejection.

Heart transplantation has improved over the last 30 years and has gradually become of increasing importance in the treatment of end-stage heart failure. Survival after transplantation has been extended with the use of immunosuppressive agents. Opportunistic infections, rejection and coronary vasculopathy in the cardiac allograft have led to the development of new immunosuppressive agents.^7^

Cyclosporine and tacrolimus have similar efficacy in protection against acute rejection in heart transplantation. However, they have similar nephrotoxicity and cardiovascular adverse events as well. Cardiac allograft coronary vasculopathy (CAV) is the best predictor of mortality five years after transplantation and accounts for 31% of deaths. Keogh found that neither cyclosporine nor tacralimus prevented the development of CAV, however, MMF did prevent CAV.^8^

With the use of immunosuppressive agents since the early 1980s, a dramatic improvement has been observed in the survival of patients who underwent solid organ transplantation. Understanding the immune mechanism causing rejection has led to the development of novel immunosuppressive agents, which are more immune specific and less toxic, and have better pharmacokinetic and higher rejection-preventing efficacy.

MMF is an organosynthetic agent. Randomised, non-blind studies have demonstrated that MMF prevented acute rejection in patients who underwent kidney, heart and liver transplantation, and it could be used in the treatment of refractory rejection. Different from tacrolimus and cyclosporine, MMF does not cause neurotoxicity or nephrotoxicity. Compared with other agents, MMF inhibits B lymphocyte proliferation and reduces smooth muscle cell proliferation, and consequently may play a key role in the treatment of chronic rejection. Studies on the use of MMF in preventing acute rejection in patients who undergo heart transplantation are ongoing.^9^

A study presented by Roche Pharmaceutical Company at the subcommittee meeting of the Antiviral Drug Advisory Committee (ADAC) comprised 650 heart transplantations, in which the combination of MMF, cyclosporine and steroids was used in 289 cases. The study found that MMF was safe and effective for the prevention of rejection in patients who underwent heart transplantation.^10^ As a result, the US Food and Drug Administration (FDA) has approved the extension of indications for MMF use for the prevention of organ rejection in patients undergoing heart transplantation.

Multiple drug therapy based on cyclosporine, steroids and azathioprine has improved the outcomes of solid organ transplantation. However, acute rejection episodes are not less common and influence short- and long-term prognosis after heart transplantation. The benefits of these agents are limited by their side effects, such as bone marrow suppression and renal dysfunction. Mathieu *et al.*^11^ retrospectively evaluated clinical and laboratory analyses obtained from 31 consecutive patients who underwent heart transplantation between 1996 and 1998 in the Montreal Heart Institute. It was found that the rejection-free period was significantly longer in the MMF group, the infection-free period was similar, and there was no difference between the groups in terms of infectious agents.

Initial studies recommended MMF because it reduces T and B lymphocyte proliferation and may decrease the frequency of acute rejection after renal transplantation. A randomised, double-blind, multi-centre, placebo-controlled study compared the reliability and efficacy of MMF. Result showed MMF was well-tolerated and significantly reduced the incidence of rejection in the six-month period after transplantation.^12^

Dipchand *et al.*^13^ retrospectively investigated patients who had undergone paediatric heart transplantation in the Hospital for Sick Children in Toronto, Canada. Of the 21 paediatric cases who received MMF, 12 were boys and nine were girls. Indication for transplantation was complex congenital heart disease in 14, cardiomyopathy in six and acute viral myocarditis in one case. The results of MMF were found to be encouraging for recipients of paediatric heart transplantation.

Rose *et al.*^14^ conducted a double-blind study comprising 86 patients from three centres. The control group consisted of 650 patients from 28 centres. The patients randomly received cyclosporine (CYC) and steroids in addition to MMF or azathioprine (AZA). The levels of anti-HLA antibodies and anti-vimentin antibodies of the patients were measured using enzyme-linked immunoassay. The basis of the study was that vimentin is the major protein of anti-endothelial antibodies and the production of anti-vimentin antibodies long term is an independent risk factor for post-transplant coronary artery disease. Mean annual anti-vimentin antibody titres were found to be significantly higher in the AZA than the MMF group.

Pharmacodynamics plays an important role in monitoring immunosuppression therapy. Previous studies have demonstrated significant correlation between pharmacodynamics, dose, and graft histology. A study investigated inhibition of lymphocyte proliferation and inhibition of expression of clusters of differentiation (CD) 134, CD71, CD11a and CD25 via flow cytometry. CYC was evaluated *in vivo* in rats, alone or in combination with MMF. Inhibition of lymphocyte function was assessed after 24 hours and found to be higher in all markers in the combination therapy compared to CYC therapy.^15^

Marcus *et al.*^16^ conducted a study to investigate the effects of CYC, FK 506 and MMF on leukocyte infiltration in grafts (over CD4, CD8, CD11a, CD18) after cardiac transplantation in rats. Transplantation was performed in 340 rats, which were divided into four groups: CYC, MMF, FK 506, and the control group not receiving immunosuppressive agent. It was observed that CYC and FK 506 decreased graft leukocyte infiltration (presence of CD4, CD8, CD11a and CD18 in the perivascular space and intra- and epicardial arteries) compared to the control group. It was determined that MMF reduced infiltration more significantly and acted earlier compared to the other two calcineurin inhibitors.

Weigel *et al.*^17^ conducted a study including 36 patients who underwent orthotopic heart transplantation. The patients were divided into two groups, AZA and MMF. Within the groups, there was no difference between the recipients in terms of age, gender, indication for transplantation, donor age, and donor ischaemic period. The control group (*n* = 15) received CYC, AZA and prednisolone. The study group (*n* = 21) received MMF instead of AZA three months after transplantation. Activation markers CD25, CD38, CD69 and human leukocyte antigen (HLA-DR) (found in B lymphocytes), T cells and natural killer (NK) cells were measured by flow cytometry.

A significant difference was observed in the reduction of B lymphocyte counts in the MMF group versus the AZA group. In addition, the percentage of CD38 B lymphocytes, activated T lymphocytes (CD4/CD25, CD8/CD38), HLA-DR and NK cells were decreased during MMF therapy. This study suggested that MMF therapy regulates the activation markers in B lymphocytes while decreasing B lymphocyte counts.

A total of 22 orthotopic heart transplantations were performed between April 1995 and February 2002 in the Onasis Cardiac Surgery Centre.^18^ Within this period, 532 patients were selected and 223 were approved for pre-transplantation. AZA, CYC and steroids were used for initial immunosuppression, with MMF used instead of AZA in 16 patients. Gradually, AZA has been completely replaced by MMF in cases that have exceeded three years post transplant. A total of 19 patients were followed for more than one year after transplantation and it was found that one (5.3%) patient died, three (15.8%) developed rejection and three (15.8%) coronary artery disease.

Klupp *et al.*^19^ divided rats that underwent allograft heart transplantation into four groups, each including six rats. Each of the groups receiving low- or high-dose MMF was divided into two subgroups. Pharmacokinetics (measured by high-performance liquid chromatography), pharmacodynamics and histological graft rejection scoring were performed in all animals on the sixth day. Rejection scoring was found to be more associated with MPA plasma concentration in terms of suppression of lymphocyte proliferation and transferrin receptor expression. Good lymphocyte suppression was provided in the low-dose group (5 mg/kg MMF BID) and ongoing pharmacodynamics were also good. No difference was found between low- and highdose groups in terms of rejection scores.

In initial clinical studies, MMF has been used instead of AZA in triple therapies. In a randomised study comprising 50 patients from 28 centres, MMF was compared with AZA in triple therapy after cardiac transplantation. It was found that the need for rejection therapy was decreased and the one-year mortality rate was substantially reduced in the MMF group.^5^

Pethig *et al.*^21^ investigated systemic inflammatory response in patients who had undergone heart transplantation and had been receiving immunosuppressive agents containing AZA or MMF. Systemic inflammatory response was found to be lower in the MMF group. High-quality, randomised studies have demonstrated that MMF, when used together with CYC and steroids, reduced the frequency and intensity of rejection and improved the grafts, in patients who underwent heart and kidney transplantation.22

A limitation of our study was that the number of study subjects was low. Also the efficacy of MMF was investigated compared with only MP, but not with a control group. Since MP is an immunosuppressive agent approved by the scientific population, it was selected instead of placebo.

## Conclusion

This study compared the effects of MMF and MP on duration of motor activity and rejection rate of transplants in rabbits that underwent retroperitoneal heterotopic heart transplantation. We found that MMF caused statistically significantly longer duration of motor activity in the transplanted hearts. No statistically significant difference was found between the MMF and MP groups in terms of transplant rejection rate. However, based on the absence of a significant difference with MP, which is a potent and important immunosuppressive agent in rescue therapy in terms of prevention of rejection, we concluded that MMF is also important for the prevention of rejection in transplantation.
